# EDTA Shuttle Effect vs. Lignosulfonate Direct Effect Providing Zn to Navy Bean Plants (*Phaseolus vulgaris L* ‘Negro Polo’) in a Calcareous Soil

**DOI:** 10.3389/fpls.2016.01767

**Published:** 2016-11-28

**Authors:** María T. Cieschi, Ana Benedicto, Lourdes Hernández-Apaolaza, Juan J. Lucena

**Affiliations:** Department of Agricultural Chemistry and Food Science, Autonomous University of MadridMadrid, Spain

**Keywords:** ZnEDTA, ^67^Zn, lignosulfonates, shuttle effect, navy beans

## Abstract

Zn-Lignosulfonates (LS) fertilizers are used as an eco-friendly alternative to chelate formulations. The mechanisms of Zn release in the rhizosphere by both types of products are compared. The ability to provide Zn to *Phaseolus vulgaris L* of non-modified and chemically modified ZnLS and ZnEDTA is compared in a hydroponic assay. Stable isotope ^67^Zn was used to study Zn source (fertilizer, Zn_Fer_, or native, Zn_Nat_) uptake and distribution in plants in two soil pot experiments. ZnEDTA was the best treatment to provide both Zn_Fer_ and Zn_Nat_ to navy bean plants. A shuttle effect mechanism and an isotopic exchange may occur. ZnLS from eucalyptus (ZnLSE) provides more Zn to the plant than LS from spruce. Chemical modifications of ZnLSE does not improve its efficiency. A double dose of ZnLSE provides similar Zn_Fer_ in leaves and similar soluble Zn_Fer_ content in soil than ZnEDTA. A model for the Zn fertilizers behavior in the soil and plant system is presented, showing the shuttle effect for the synthetic chelate and the direct delivery in the rhizosphere for the ZnLS complex.

## Introduction

Zinc (Zn) deficiency is one of the most widespread micronutrient disorders among different crops (Westfall et al., 1971; Romheld and Marschner, 1991) affecting the quantity and the nutritional quality of the harvest (Cakmak et al., 1997). This fact is of special importance because Zn deficiency in humans has a high prevalence in the world (an estimated 31% in the global population). In total, 1.4% (0.8 million) of deaths worldwide were attributable to zinc deficiency: 1.4% in males and 1.5% in females (World Health Organization, 2002).

Zinc synthetic chelates such as ZnEDTA are the common source used to alleviate Zn deficiency of crops. Synthetic chelates application, regarded as the most effective source of plant micronutrients (Alloway, 2008) is limited to cash crops because of its high price. Synthetic chelating agents are so persistent in the environment that they can largely contribute to accelerating the movement of Zn and other metals (Álvarez et al., 1996; Li and Shuman, 1997; Rodella and Chiou, 2009) in soil profiles that may produce accumulations of trace elements in ground water and over the years, Zn deficiency (Mikkelson and Brandon, 1975). Zinc natural organic complexes such as Zn-Lignosulfonate (ZnLS) forms another group of Zn fertilizers that are more biodegradable and less expensive than synthetic chelates because they are by-products from the manufacturing of paper pulp through sulphite process. Lignin polymers became water-soluble by the introduction of sulfonic acid groups. The presence of a considerable number of anionic groups including sulfonic, carboxylic acid and phenolic hydroxyl groups (Askvik et al., 1999) which can form coordinated bonds with metals converts the LS into an appropriate micronutrient’s complexing agent. In addition, the utilization of LS prevents the environmental impact of the black liquor of the paper industry due to the LS removed from it (González et al., 1989). Composition and properties of LS depend on the kind of wood source, the procedure used to obtain the paper pulp and the process of treatment of the black liquor. Therefore, the composition of the final product presents a wide variability. In order to improve properties required in final applications and to reduce the impurity content, the LS are treated physical and chemically. It is important to understand the relationship between the modification reactions and the characterization of LS. The molecular weight, the presence and the amount of functional groups could modify the efficacy of the fertilizers based on LS as a complexing agent. In a previous work, Benedicto et al. (2011) studied Zn Complexing Capacity (ZnCC) of six ZnLS complexes employed in this work and their reactivity vs. pH. This work continues the physical-chemical characterization of six LS complexes, by studying their reactivity vs. different soil components.

In 2015, more than 25% of the Zn complexes sold in the Spanish market used the LS as the complexing agent (Sánchez Jiménez and Lucena, 2015). European regulation allows its application in fertirrigation, by foliar spray and recently, direct application to soil. Several authors tested the efficiency of ZnLS complexes in hydroponic culture conditions, in soil conditions or by foliar applications (Álvarez et al., 1996; Moraghan, 1996; Gangloff et al., 2002; Álvarez-Fernández et al., 2007; Gonzalez et al., 2008; Martín-Ortiz et al., 2009; Benedicto et al., 2011).

In most cases the LS are less effective (Mortvedt and Gilkes, 1993) than chelates because of its known weak bonds and low complex stability. Lindsay and Schwab (1982) proposed a mechanism for the action of iron chelates in soils in which the chelating agent works ideally as a shuttle iron transporter between soil and plant roots. This mechanism was later referred to as the “shuttle effect” (Lucena, 2003) but nothing has been yet demonstrated. An attempt to explain zinc chelates and zinc lignosulfonates (LS) reactions in soil, iron theoretical model (Lucena, 2006) was adapted for Zn in this work.

At the present time, ^67^Zn is a tracer tool to evaluate Zn uptake and distribution behavior in plants (Benedicto et al., 2011; McBeath et al., 2013). The ^67^Zn is a natural stable isotope (abundance: 4.1%), so it can be safely used with high flexibility in experimental designs and field studies. Moreover, zinc isotope amounts can be quantified by ICP-MS, and after mathematical deconvolution (Benedicto et al., 2011), Zn from the fertilizers (Zn_Fer_) and Zn from natural sources such as the soil or the seeds (Zn_Nat_) can be determinated in each plant organ or soil fraction. Schenkeveld et al. (2014) suggested that tracer studies with isotopically labeled iron chelates would provide more understanding of the actual effectiveness of the shuttle mechanism in soil-plant systems. In this way, the theoretical iron model would be renewed and adjusted.

The aims of this work were first, to compare modified and non-modified ZnLS complexes efficiency in respect to ZnEDTA in hydroponics and soil conditions, and secondly, to evaluate EDTA shuttle effect and ZnLS complexes direct effect in soil and their influence in navy bean zinc nutrition.

## Materials and Methods

### Lignosulfonates

Six LS, kindly provided by Lignotech Iberia S.A. (Torrelavega, Spain), were applied in this study. They were obtained through sulfite treatment of hardwood (eucalyptus; LSE) and softwood (Spruce; LSS) sources. LSEO, LSES, LSEP were obtained from LSE by industrial modifications: oxidation, sulfonation and phenolation respectively, in order to increase the amount of functional groups of the polymer capable of complexing Zn. LSEU was obtained through ultrafiltration with the objective of reducing the molecular weight of the polymers. The LS here studied, were previously described elsewhere (Benedicto et al., 2011; Rodríguez-Lucena et al., 2011). Main chemical characteristics of products are shown in **Table 1**.

**Table 1:** Chemical characteristics of the products.

	LSS	LSE	LSEO	LSES	LSEP	LSEU
Dry content (%)	50.3	53.7	48.1	48.6	14.0	46.8
Average Molecular weight (g.mol^-1^)	25732	6275	7550	7903	6259	4975
LS content (g LS Kg^-1^ dw)	835	587	559	653	504	390
Maximum complexing capacity (gZn Kg^-1^LS)	162	206	291	252	371	375
pH	3.6	4.3	6.8	4.7	3.5	2.3
Organic-S (g⋅Kg^-1^ dw)	55	51	45	57	45	55
Phenolic-OH (g⋅Kg^-1^ dw)	19	19	18	18	31	18
-COOH (g⋅Kg^-1^ dw)	26	35	77	67	63	58

### Zn Treatments for Hydroponic Experiment

The ZnLS complexes were formed by mixing a solution of ZnSO_4_⋅ H_2_O (Merck) and the suitable amount of LS to complex Zn, calculated based on their maximum complexing capacity (**Table 1**). In order to ensure that all the quantity of element is complexed, an amount of 10% LS was added in excess. Solutions were left to stand overnight, filtered through 0.45 μm Millipore membrane and made up to volume.

For the preparation of the ZnEDTA solution, Na_2_EDTA (Merck) was dissolved in sufficient NaOH (1:3 molar ratio) and an amount of ZnSO_4_⋅ H_2_O (Merck) calculated to be 5% in excess of the molar amount of ligand was then slowly added. During the chelation process, pH was maintained between 6.0 and 8.0. The solution was left to stand overnight, filtered through 0.45 μm Millipore membrane and made up to volume.

### Efficacy in Hydroponics

Navy bean (*Phaseolous vulgaris* L. c.v. Negro Polo) seeds, which are sensitive plants to zinc deficiency, were germinated in the dark at 30°C on filter paper moistened with distilled water. After germination, seedlings were transferred to a Dycometal-type CCK growth chamber where they grew until the end of the experiment under controlled climatic conditions: day/night photoperiod, 16/8 h; temperature (day/night) 30/25°C, relative humidity (day/night) 50/70%. The composition of the Zn-free nutrient solution was the following: (macronutrients in mM) 1.0 Ca(NO_3_)_2_, 0.9 KNO_3_, 0.3 MgSO_4_, 0.1 KH_2_PO_4_; (cationic micronutrients in μM as buffered micronutrient solution) 2.5 MnSO_4_, 1.0 CuSO_4_, 1.0 NiCl_2_, 1.0 CoCl_2_, 105.5 Na_2_EDTA, 20.0 FeEDDHA; (anionic micronutrients in μM) 35.0 NaCl, 10.0 H_3_BO_3_, 0.05 Na_2_MoO_4_. The nutrient solution during all of the experiments was continuously aerated, and the pH was buffered with 1.0 × 10^-4^ M HEPES and adjusted at 7.5–8.0 with 1.0M KOH. First, seedlings were placed on containers filled with 1/5 diluted nutrient solution with a concentration of 5 μM Zn as ZnEDTA. After 5 days, the diluted nutrient solution was replaced by the full-strength Zn-free nutrient solution. Seedlings grew in this solution for 6 days to induce Zn deficiency. Then the plants were transferred to polyethylene pots (three pairs of plants per pot) containing 2 L of the full-strength Zn-free nutrient solution except for micronutrient content (not buffered micronutrient solution, in μM): 1.0 MnSO_4_, 0.5 CuSO_4_, 0.1 NiCl_2_, 0.1 CoCl_2_, and 20.0 FeEDDHA. In order to simulate calcareous conditions, CaCO_3_ (0.1 g L^-1^) was added. At this time, the treatments (ZnEDTA and the ZnLS tested), at a dose of 0.5 μM per pot of Zn were applied. The nutrient solution and the treatments were renewed weekly. Four replicates (four pots) per treatment were used. Two pairs of plants were harvested at the first sampling, which was carried out at 7 days after treatments (DAT), and the second sampling at 21 DAT (the remaining plants).

### Efficacy in Soil Applications

#### Reactivity of ZnLS Complexes vs. Soil Components

Two milliliters of ZnLS complexes solution (50 mg Zn⋅L^-1^) were added to 8 mL of 12.5 mM HEPES, 12.5 mM CaCl_2_ solution and to different soil components: 0.1 g of peat, 0.1 g of illite, 0.1 g of Ca-montmorillonite, 0.8 g of calcium carbonate and 0.8 g of dolomite. Details about soil materials are described elsewhere (Álvarez-Fernández et al., 2001). Three replicates per each soil component were done. After 1 h of orbital shaking at 56 min^-1^, samples were allowed to interact for 3 days at 25°C.

All solutions were filtered through 0.45 μm Millipore membranes and the pH was measured with an Orion Research (Ion Analyzer EA920). Zn concentration in the filtrate was determined by atomic absorption spectrometry (AAS) with a Perkin Elmer Analyst 800 spectrophotometer.

### ^67^Zn Treatments

Labeled treatments (^67^ZnLS and ^67^ZnEDTA) were prepared in a Zn concentration of 420 μM by using ^67^Zn provided by Isoflex with the following isotopic distribution (atom %): ^64^Zn (1.56), ^66^Zn (3.88), ^67^Zn (89.6), ^68^Zn (4.91) and ^70^Zn (0.05). The ^67^Zn-enriched Zn was dissolved in H_2_SO_4_ Suprapur (Merck). The ^67^ZnLS were prepared by mixing ^67^Zn-enriched Zn with the suitable amount of LS to complex it, based on their maximum complexing capacity (**Table 1**). To ensure that all Zn added was complexed, a 10% extra amount of LS was added. For the preparation of the ^67^ZnEDTA solution, an amount of ^67^Zn-enriched Zn was slowly added to a solution of Na_2_EDTA (as Tritiplex III, Merck). In order to chelate all ^67^Zn, Na_2_EDTA was calculated 5% in excess. During the chelation process, the pH was maintained between 6.0 and 8.0. Finally, the pH of all solutions was adjusted up to 6.0 with 0.1M NaOH. Then, treatments were left to stand overnight, filtered through a 0.45 μm Millipore membrane, and made up to volume.

### Soil Pot Experiments

#### Comparison between Treatments

Five ^67^Zn treatments (2.3 μmol ^67^Zn per pot) were applied over soil surface: ZnEDTA, ZnLSS, ZnLSE and ZnLSEO. In addition, a control without Zn was assayed.

Navy bean seeds were germinated and Zn deficiency was induced as described in the hydroponic assay but the iron source used in nutrient solution was FeHBED 50 μM. Seedlings were transferred (three plants per pot) after 8 days to 600 g polystyrene pots filled of a soil/sand 70/30% (w/w) mixture. The soil was obtained from the first 20 cm of a citrus farm at Picassent, Valencia, Spain (39°21′41.28″ N, 0°27′ 42.58″ W). Physicochemical characteristics of this soil are described in **Table 2**. Texture, pH, soil electrical conductivity (E.C), soil organic matter (OM), C/N ratio, CaCO_3_ were measured according to the official methods (Ministerio de Agricultura, Pesca y Alimentacion, 1994) and micronutrients availability as in Soltanpour and Schwab (1977). Normalized calcareous sand (2–4 mm) was used. Before transferring the seedlings, pots were irrigated till field capacity. Then, pots were covered with foil to avoid the chelate photodegradation and algae development (Hill-Cottingham, 1955; Hernández-Apaolaza and Lucena, 2011). Once the seedlings were transferred (three plants per pot), the pots were watered daily with macronutrient solution (in mM): 0.8 Ca(NO_3_)_2_, 0.9 KNO_3_, 0.2 MgNO_3_, 0.1 KH_2_PO_4_ and micronutrient solution (in μM): 2.5 MnSO_4_, 1.0 CuSO_4_, 1.0 NiCl_2_, 1.0 CoSO_4_, 50.0 FeHBED. Both solutions were saturated with CaCO_3_ (0.1 g L^-1^) to simulate calcareous conditions. Five replicates (five pots) per treatment were done. Two samplings were carried out at 7 DAT and 21 DAT. At first sampling two plants were harvested and at the second sampling, the remaining one.

**Table 2:** Physical and chemical properties of the soil used for both soil experiments.

Sand (g⋅Kg^-1^)	435
Silt (g⋅Kg^-1^)	80
Clay (g⋅Kg^-1^)	485
pH (H_2_O)	7.9
E.C._1:5_ (dS m^-1^)	0.2
OM (g Kg^-1^)	9.2
N Kjeldahl (g Kg^-1^)	0.3
C/N	30.7
CaCO_3_ (g⋅Kg^-1^)	380
CaCO_3_ active (g⋅Kg^-1^)	89
DTPA Zn (mg⋅Kg^-1^)	3.00
DTPA Fe (mg⋅Kg^-1^)	5.3
DTPA Mn (mg⋅Kg^-1^)	4.5
DTPA Cu (mg⋅Kg^-1^)	1.1

#### Comparison between Doses

A similar assay to the previous one was done, but only one plant per pot and one sampling time at 21 DAT, days after treatments, were done. As in the previous pot experiment, the soil belonged to the first 20 cm of a citrus farm at Picassent, Valencia, Spain. In this case, three doses of ZnLSS (ZnLSS1, ZnLSS2 and ZnLSS3) and ZnLSE (ZnLSE1, ZnLSE2 and ZnLSE3) were applied: 1.5, 2.3, and 4.6 μmol ^67^Zn per pot over the soil surface and were compared to one dose of ZnEDTA: 2.3 μmol ^67^Zn per pot. Pots without plants were also prepared in order to study the plant effect in soil reactions.

### Analytical Procedures

The sampled roots, stems and leaves were separated, weighed and washed with 0.1% HCl and 0.01% non-ionic detergent (Tween 80) solution and rinsed with ultrapure water. Then samples were dried in a forced air oven at 65°C for 3 days. Plant samples were mineralized by microwave (CEM Corporation MARS 240/50; Mathews, NC, USA) digestion with HNO_3_ 65%, H_2_O_2_ 30% and one drop of HF 40%. Total zinc was measured using Atomic Absorption Spectrophotometry (AAS) for the hydroponic and interaction experiment.

Soil soluble fraction was obtained by washing the soil with distilled water (600 mL) and stirred for 10 min with a rotary stirrer at 90 min^-1^. An aliquot of 40 mL was centrifuged for 5 min at 6000 min^-1^ (Rotofix 32 Hettich), the supernatant was filtered by Filter Lab 1238 and then, filtered by Millipore 0.45 μm syringe filter. HNO_3_ (Suprapur, Merck) was added up to achieve a 1% acid matrix.

Soil available fraction were obtained from the remaining solid in the centrifuge tube by extraction for 20 min with 25 mL of Soltanpour and Schwab (1977) extractant (DTPA + ammonium bicarbonate). After that, the samples were filtered. The extraction was made in triplicate, the extracts were joined in a single extract, and volume amounted up to 100 ml. Nitric acid was added to eliminate the excess of bicarbonate and to allow an acid media for the analytical determinations.

Isotope quantification in the plant organs and soil fractions (soluble and available) were determined by ICP-MS (7500c, Agilent Technologies, Santa Clara, CA, USA). Zinc from the fertilizers (Zn_Fer_) and Zn from natural sources as soil or seeds (Zn_Nat_) were calculated by isotope pattern deconvolution analysis (Rodríguez-Castrillón et al., 2008; Benedicto et al., 2011) in the biological soil experiments. In brief, the mass balance for all the Zn natural isotopes can be expressed as shown by matrix notation:

$$\left[\begin{array}{c} {}^{64}A_{Total}\\ {}^{66}A_{Total}\\ {}^{67}A_{Total}\\ {}^{68}A_{Total} \end{array}\right]=\left[\begin{array}{c} {}^{64}A_{Fer}\\ {}^{66}A_{Fer}\\ {}^{67}A_{Fer}\\ {}^{68}A_{Fer} \end{array}\begin{array}{c} {}^{64}A_{Nat}\\ {}^{66}A_{Nat}\\ {}^{67}A_{Nat}\\ {}^{68}A_{Nat} \end{array}\right]\times \left[\begin{array}{c} x_{Fer}\\ x_{Nat} \end{array}\right]+\left[\begin{array}{c} {}^{64}e\\ {}^{66}e\\ {}^{67}e\\ {}^{68}e \end{array}\right]$$

Where each A_Total_, is the isotope abundance of each Zn isotope in the vegetal sample. A_Fer_ is the corresponding isotope abundance in the tracer, and A_Nat_ is the natural isotope abundance. Moreover, x_Fer_ and x_Nat_ denote the molar fractions of Zn in the isotopically altered sample arising from the two different sources of the element (fertilizer or natural). The best values of x_Nat_ and x_Fer_ are found by least-squares fitting of the error vector *e* (minimizing the square sum of errors) using the SOLVER application in Excel^®^ (Benedicto et al., 2011).

### Statistical Analysis

In order to verify the homogeneity of the data, the Levene test was first used. Then, differences between Zn treatments were tested for significance by one- or two-way analysis of variance (ANOVA) as appropriate. Means were compared using the Duncan multiple range test (*P* < 0.05). Results of two way ANOVA are expressed as ns (not significant), ^∗^*P* < 0.05, ^∗∗^*P* < 0.01 and ^∗∗∗^*P* < 0.001. Using SPSS v. 21.0 software, all calculations were performed.

## Results

### Efficacy in Hydroponics

Four modified (ZnLSEO, ZnLSES, ZnLSEP, ZnLSEU) and two non-modified ZnLS complexes (ZnLSS and ZnLSE) were tested and compared to ZnEDTA and Control without Zn. Data obtained in the first sampling (7 DAT) is not shown because statistical differences were scarce. **Table 3** presents total Zn (μg g^-1^) in leaves of navy bean plants 21 DAT. Leaf dry weight ranged from 2.61 g pot^-1^ (ZnLSES) to 3.12 g pot^-1^ (ZnEDTA) and didn’t show significance differences between the treatments (Supplementary Table S1). Zinc amounts were higher in plants treated with ZnLS complexes than in Control plants and over the deficiency threshold of 20-μg g^-1^ (Jones and Mills, 1991), which indicates that ZnLS complexes are able to provide Zn to navy bean plants applied in nutrient solution. ZnEDTA and ZnLS complexes did not present significant differences in respect to dry weight and Zn concentration in leaves; thereby ZnLS complexes were capable of supplying Zn to navy bean plants similarly to ZnEDTA in these experimental conditions.

**Table 3:** Zn_Total_ in leaves for hydroponic experiment at 21 DAT.

Treatments	Zn_Total_ (μg g^-1^)
Control	15.1 ± 0.89^b^
ZnLSS	32.8 ± 1.17^a^
ZnLSE	33.6 ± 1.96^a^
ZnLSEO	33.0 ± 3.23^a^
ZnLSES	32.8 ± 2.27^a^
ZnLSEP	32.4 ± 1.90^a^
ZnLSEU	33.0 ± 3.92^a^
ZnEDTA	30.4 ± 2.11^a^

*In each data range, different letters denote significant differences among the treatments according to Duncan’s test (*P* < 0.05). ns, not significant.*

### Efficacy in Soil Applications

#### Reactivity of ZnLS Complexes vs. Soil Components

**FIGURE 1:**
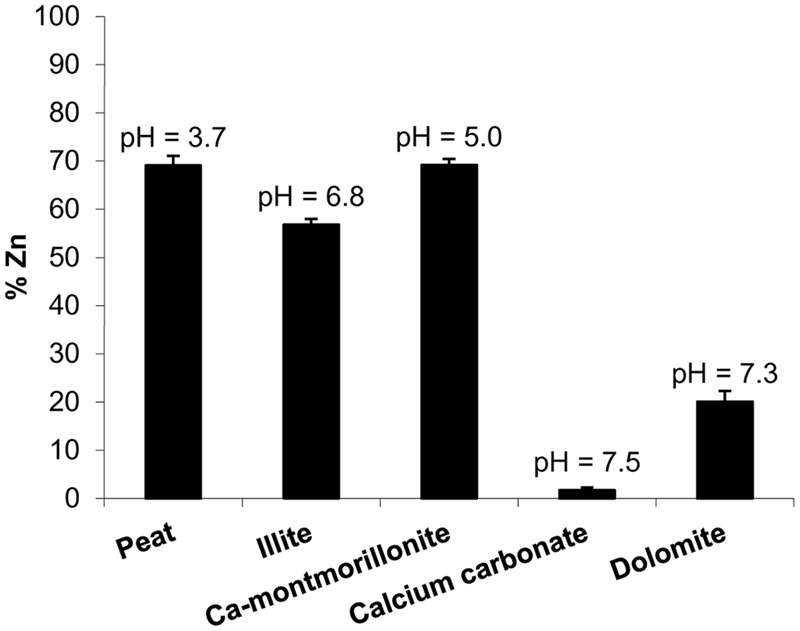
**Zn percentage remained in solution after 3 days of interaction of lignosulfonates with different soil components (peat, illite, Ca.-montmorillonite, calcium carbonate, dolomite) and final pH observed**.

According to the bifactorial statistical analysis, (soil material and zinc source as factors) differences among sources were scarce. Averages for all soil components show that for ZnLSEP and ZnLSE the 44.0% of the Zn remained in solution and are slightly less reactive than ZnLSES (less than 42.6% Zn remained in solution). Differences among soil materials were more important than differences between LS. **Figure 1** presents the percentage of the Zn remained in solution after 3 days of interaction of every ZnLS treatment with each soil component and the final pH. **Figure 1** is a summary of the reactivity of the LS treatments studied because they did not presented significant differences between them. In general, around 30% of the Zn added with ZnLS complexes remained with peat and Ca-montmorillonite so they were not in solution despite reaching acid pH. The loss of soluble Zn was slightly higher with illite with pH values near to the neutrality. However, with dolomite only around 20% of the Zn remained in solution at pH 7.3 and almost the total Zn added as ZnLS complexes was lost in the solution after interaction with CaCO_3_.

### Soil Pot Experiments

Zinc amounts in leaves are related to the efficacy of the treatments. **Tables 4** and **5** show, Zn_Fer_ and Zn_Nat_ contents (μmol pot^-1^) and Zn_Total_ (μg g^-1^), calculated from the molar amounts and using the Zn molecular weight considering natural distribution of the isotopes in navy bean leaves for the first and second soil experiment after 3 weeks of treatments (21 DAT). According to Jones and Mills (1991) the navy bean plants are sufficiently zinc nourished when the total zinc concentration in leaves is over 20 μg g^-1^. The Zn_Total_ concentrations ranged from 14.9 μg g^-1^ (control) to 26.0 μg g^-1^ (ZnEDTA) in the first pot experiment and from 25.9 μg g^-1^ (control) to 54.3 μg g^-1^ (ZnEDTA) in the second experiment. In the first pot experiment, the zinc deficiency was corrected after applying the treatments and in the second experiment, the navy bean plants presented an adequate nutrition.

**Table 4:** Zn_Fer_ and Zn_Nat_ (μmol pot^-1^) and Zn_Total_ (μg g^-1^) in leaves for the first soil experiment at 21 DAT.

Treatments	Zn_Fer_ (μmol pot^-1^)	Zn_Nat_ (μmol pot^-1^)	Zn_Total_ (μg g^-1^)
Control	–	0.46 ± 0.04^b^	14.9 ± 0.43^b^
ZnLSS	0.10 ± 0.02^ab^	0.48 ± 0.02^b^	18.4 ± 1.60^bc^
ZnLSE	0.09 ± 0.02^b^	0.51 ± 0.04^b^	18.2 ± 1.35^b^
ZnLSEO	0.06 ± 0.02^b^	0.54 ± 0.03^ab^	17.7 ± 1.33^bc^
ZnEDTA	0.18 ± 0.03^a^	0.67 ± 0.12^a^	26.0 ± 1.20^a^

*In each data range, different letters denote significant differences among the treatments according to Duncan’s test (*P* < 0.05). ns, not significant. ^∗^Total Zn was calculated using the Zn molecular weight considering natural distribution of the isotopes.*

**Table 5:** Zn_Total_ (μg g^-1^) in leaves for the second soil experiment at 21 DAT.

Treatments	Zn_Total_ (μg g^-1^)^∗^
Control	25.9 ± 1.95^b^
ZnLSE1	31.6 ± 2.72^b^
ZnLSE2	36.6 ± 6.69^b^
ZnLSE3	37.2 ± 3.02^b^
ZnLSS1	34.6 ± 2.77^b^
ZnLSS2	37.3 ± 3.11^b^
ZnLSS3	31.3 ± 3.91^b^
ZnEDTA	54.3 ± 3.16^a^

*In each data range, different letters denote significant differences among the treatments according to Duncan’s test (*P* < 0.05). ns, not significant. ^∗^Total Zn was calculated using the Zn molecular weight considering natural distribution of the isotopes.*

In the first soil experiment the two non-modified (ZnLSS and ZnLSE) and one modified (ZnLSEO) zinc LS treatments were tested and compared with ZnEDTA and Control without Zn. All treatments were prepared with labeled ^67^Zn. Data obtained in the first sampling (7 DAT) are not shown because statistical differences were not found.

Navy bean leaves dry weight ranged from 2.03 g pot^-1^ (control) to 2.27 g pot^-1^ (ZnLSEO) and did not show significant differences between the treatments applied for the first soil pot experiment at 21 DAT (Supplementary Table S2). Navy bean plants treated with ZnEDTA presented the highest Zn_Total_ (**Table 4**). ZnEDTA and ZnLSS were the treatments that provided the highest Zn_Fer_ content to the plant. Moreover, total zinc amounts were higher in plants treated with ZnLS complexes than in Control plants and there were not significant differences between lignosulfonate treatments.

In the second soil experiment different doses of ZnLS complexes (1.5, 2.3 or 4.6 μmol ^67^Zn per pot) were applied and were compared to ZnEDTA (2.3 μmol ^67^Zn per pot). **Table 5** presents navy bean leaf dry weight ranged from 1.23 g pot^-1^ (ZnLSE1) to 1.48 g pot^-1^ (ZnEDTA). Navy bean plants treated with ZnEDTA and ZnLSA1 presented the highest dry weight (Supplementary Table S3).

**FIGURE 2:**
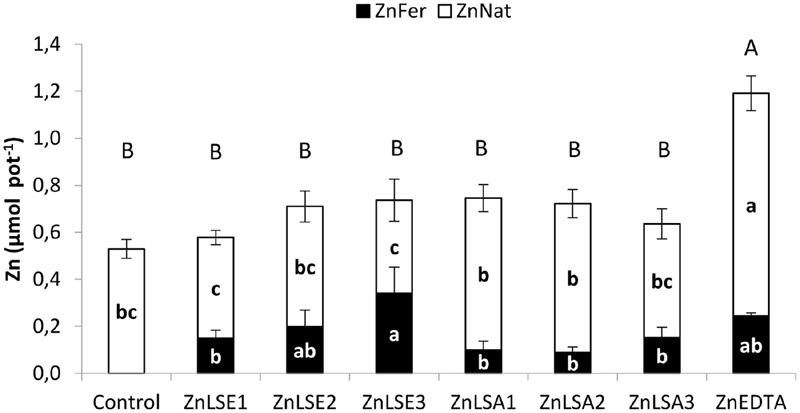
**Zn_Fer_ and Zn_Nat_ (μmol pot^-1^) content in navy bean leaves after 3 weeks of treatments.** For each series different letters denote significant differences among the treatments according to Duncan’s Test (*p* < 0.05). Lowercase letters correspond to Zn_Fer_ and Zn_Nat_ and capital letters correspond to Zn_Total_ statistical results.

**Figure 2** shows Zn_Fer_ and Zn_Nat_ contents in leaves (μmol plant^-1^) at 21 DAT. **Table 6** shows the bifactorial statistical analysis which evaluated the effects of doses and treatments on leaf dry weight, and contents of Zn_Fer_, Zn_Nat_ and Zn_Total_ (μmol plant^-1^) in leaves, soluble and available soil fractions with or without plant, between ZnLS. From now on, in this paper, Zn_Total_ is the result of (Zn_Fer_ + Zn_Nat_) since is expressed in units of contents of zinc (μmol plant^-1^).

**Table 6:** Effect of doses (D) and treatments (T) related to the contents of Zn_Fer_, Zn_Nat_ and Zn_Total_ (μmol pot^-1^) in navy bean leaves, soluble (SF) and available (AF) soil fractions with and without plants.

					Treatments		Doses (μmol pot^-1^)		
		D	T	DxT	ZnLSE	ZnLSS	1.5	2.3	4.6
Leaves	Zn_Fer_	^∗^	^∗^	ns	0.23^a^	0.11^b^	0.13^b^	0.14^b^	0.25^a^
	Zn_Nat_	^∗^	^∗^	ns	0.44^b^	0.59^a^	0.54^ab^	0.57^a^	0.44^b^
	Zn_Total_	ns	ns	ns	0.67^ns^	0.70	0.66^ns^	0.69	0.72
SF with plants	Zn_Fer_	^∗∗∗^	ns	ns	0.11^ns^	0.11	0.06^c^	0.10^b^	0.18^a^
	Zn_Nat_	ns	ns	ns	0.52^ns^	0.60	0.53^ab^	0.49^b^	0.65^a^
	Zn_Total_	^∗^	ns	ns	0.63^ns^	0.72	0.59^b^	0.59^b^	0.83^a^
AF with plants	Zn_Fer_	^∗∗∗^	ns	ns	1.95^nsc^	2.13	0.89^c^	1.70^b^	3.54^a^
	Zn_Nat_	ns	ns	ns	10.2^ns^	10.5	10.4^ns^	10.2	10.4
	Zn_Total_	^∗∗∗^	ns	ns	12.1^ns^	12.6	11.3^b^	11.9^b^	14.0^a^
SF without plants	Zn_Fer_	^∗∗∗^	ns	ns	0.03^ns^	0.03	0.02^b^	0.02^ab^	0.05^a^
	Zn_Nat_	ns	^∗^	ns	0.20^b^	0.26^a^	0.27^a^	0.19^b^	0.23^ab^
	Zn_Total_	ns	ns	ns	0.22^ns^	0.29	0.27^ns^	0.21	0.28
AF without plants	Zn_Fer_	^∗^	ns	ns	2.77^ns^	2.77	1.38^c^	2.67^b^	4.27^a^
	Zn_Nat_	ns	ns	^∗^	16.0^ns^	14.4	14.6^ns^	16.0	14.9
	Zn_Total_	^∗^	ns	ns	18.8^ns^	17.1	16.0^b^	18.7^a^	19.1^a^

*Means in the same row followed by the same letter do not differ significantly according to the Duncan test (*P* < 0.05). Two way ANOVA results. ^∗^*P* < 0.05, ^∗∗∗^*P* < 0.001; ns, not significant. SF, soluble soil fraction. AF, available soil fraction.*

Navy bean plants treated with ZnEDTA showed the highest content of Zn_Total_ in leaves as in the first soil study (**Figure 2**) ZnEDTA and ZnLSA1 presented the highest dry weight (**Table 5**). Regarding LS, all the treatments presented higher amounts of Zn_Total_ than plants without zinc addition and did not reveal significant differences between them. Bifactorial statistical analysis did not show an effect of doses or treatment on Zn_Total_ contents in leaves treated with ZnLS complexes (**Table 6**).

ZnEDTA, ZnLSE3 and ZnLSE2 were the most efficient treatments in providing Zn_Fer_ to the navy bean plants (**Figure 2**). Both LS showed an increasing tendency of Zn_Fer_ in leaves with the doses but it was the biggest for ZnLSE treatments. Bifactorial statistical analysis (**Table 6**) showed ZnLSE was more efficient than ZnLSS providing Zn_Fer_ to the navy bean leaves and the third dose is the most effective.

Moreover, ZnEDTA showed the highest Zn_Nat_ content in leaves (**Figure 2**), showing a similar behavior to the first soil experiment (**Table 4**). Comparison between LS in respect to Zn_Nat_ by the bifactorial statistical analysis in leaves shows ZnLSS is more efficient than ZnLSE and it depends on the doses (**Table 6**).

**FIGURE 3:**
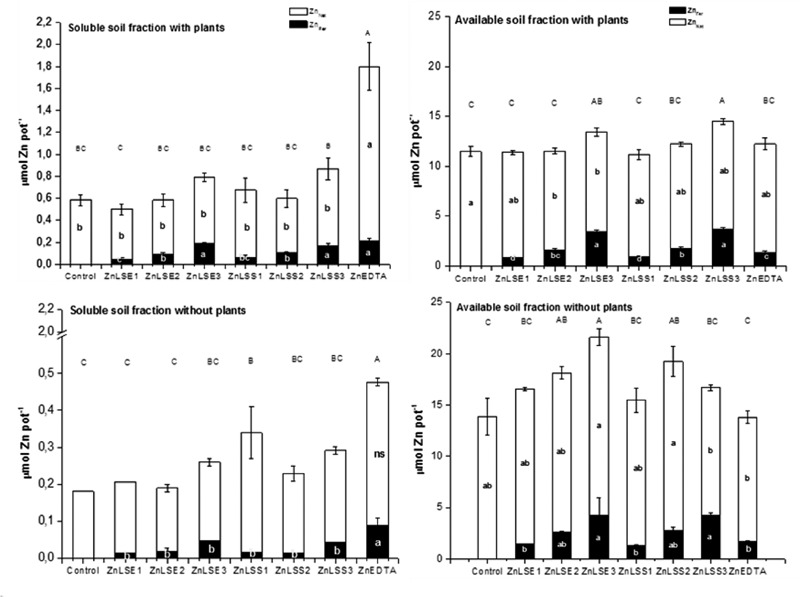
**Zn_Fer_ and Zn_Nat_ (μmol pot^-1^) content in soluble and available soil fractions in pots with plant **(top)** or pots without plant **(bottom)** after 3 weeks of treatments.** For each series different letters denote significant differences among the treatments according to Duncan’s Test (*P* < 0.05). ns, not significant. Lowercase letters correspond to Zn_Fer_ and Zn_Nat_ and capital letters correspond to Zn_Total_ statistical results.

**Figure 3** shows Zn_Fer_ and Zn_Nat_ (μmol pot^-1^) content in soluble and available soil fractions in pots with plant or pots without plant at 21 DAT. ZnEDTA presented the highest content of Zn_Total_ in soluble fraction for pots with and without plants. LS presented an increase of soluble Zn_Fer_ content with the doses for pots with and without plants. There were not significant differences between the third dose of ZnLSE or ZnLSS and ZnEDTA in pots with plants. Hence, while Zn_Total_ content for ZnLS in soluble soil fraction was less than ZnEDTA, Zn_Fer_ content was similar to ZnEDTA for the third dose. Based on the bifactorial statistical analysis (**Table 6**), a double dose of spruce or eucalyptus LS would provide similar effects to soluble Zn_Fer_ content in soil with plants.

Pots with and without plants presented similar trends in respect to Zn_Fer_ content in soil fractions. The ZnEDTA presented the highest content of Zn_Fer_ in soluble fraction and LS presented an increase of soluble Zn_Fer_ content with the doses but unlike pots with plants, Zn_Fer_ content for the third dose was lower than Zn_Fer_ for ZnEDTA.

With respect to available Zn_Fer_ contents for ZnLS, it seems to have a tendency to increase in pots with and without plants in response to the increasing dose. Furthermore, the highest zinc lignosulfonate dose presented larger availability than ZnEDTA independently of wood type (**Figure 3**).

Since one of the aims of this work was to evaluate the efficacy of lignosulfonate to provide zinc to navy bean plants, Zn_Fer_ percentage distribution in plant and soil was calculated (**Table 7**). Zn_Fer_ Percentage was calculated applying the following equation:

**Table 7:** Zn_Fer_ percentage (%) in plant and soil fractions.

	Leaf	Stem	Root	Soil available fraction	Soil soluble fraction
ZnLSE1	11 ± 2.2^a^	4.9 ± 1.0^ab^	18 ± 4.7^ns^	63 ± 7.5^bc^	3.5 ± 0.5^b^
ZnLSE2	8.4 ± 2.7^ab^	3.6 ± 0.9^ab^	10 ± 5.9	74 ± 8.0^abc^	4.2 ± 0.5^b^
ZnLSE3	7.3 ± 2.2^ab^	3.2 ± 0.9^ab^	10 ± 3.1	75 ± 5.8^abc^	4.1 ± 0.2^b^
ZnLSS1	7.4 ± 2.5^ab^	3.9 ± 1.1^ab^	11 ± 4.6	73 ± 7.6^abc^	5.1 ± 1.5^b^
ZnLSS2	3.9 ± 1.0^b^	2.5 ± 0.5^b^	8.9 ± 3.1	80 ± 4.6^ab^	4.7 ± 0.3^b^
ZnLSS3	3.5 ± 1.0^b^	2.3 ± 0.7^b^	7.0 ± 2.6	84 ± 3.8^a^	3.8 ± 0.5^b^
ZnEDTA	12 ± 0.3^a^	5.6 ± 0.6^a^	13 ± 0.9	61 ± 1.1^c^	9.2 ± 1.1^a^

*For each series different letters denote significant differences among the treatments according to Duncan’s Test (*P* < 0.05). ns, not significant.*

%ZnFer=ZnFer(μmolpot−1)Total ZnFer(μmolpot-1)×100

where, theTotal Zn_Fer_(μmol pot^-1^) = Zn_Fer_ in leaf (μmol pot^-1^) + Zn_Fer_ in stem (μmol pot^-1^) + Zn_Fer_ in root (μmol pot^-1^) + Zn_Fer_ in soil available fraction (μmol pot^-1^) + Zn_Fer_ in soil soluble fraction (μmol pot^-1^). The highest Zn_Fer_ content in plant was for ZnLSE1 and mostly located in roots (18%) due to zinc is a slightly mobile micronutrient. ZnLSS3 presented the highest Zn_Fer_ content in soil, especially in the available fraction (around 84%). ZnLSE1, ZnLSE2, ZnLSE3, ZnLSS1, and ZnEDTA showed similar Zn_Fer_ percentage for stems and leaves while all ZnLS treatments except ZnLSE1 presented the highest Zn_Fer_ percentage for the available soil fraction. ZnEDTA, as expected, was the highest treatment that soluble Zn_Fer_ percentage has provided.

A bifactorial statistical analysis between zinc lignosulfonate treatments (data not shown) revealed that Zn_Fer_ distribution in plant depends on the treatment applied. LSE was more efficient than LSS in providing Zn_Fer_ to the navy bean plants. Both treatments showed a decreasing tendency of Zn_Fer_ percentage with the doses but there were no significant differences between them. Contrary to plant results, Zn_Fer_ distribution in soil showed to be independent from the treatment (eucalyptus or spruce wood) but dependent from the doses. Available and soluble fractions presented an increasing Zn_Fer_ percentage tendency with the doses.

Iron, copper, manganese, and phosphorus were measured in plants and soil fractions in order to study the influence of zinc nutrition in the behavior of others micronutrients. Results obtained did not show disorders in micronutrients nutrition according to Jones and Mills (1991) for navy bean plants. In general, navy bean plants treated with LS presented a decrease in their phosphorous nutrition (Supplementary Tables S4–S8).

## Discussion

The hydroponic experiment showed that Zn amounts in plants treated with ZnLS complexes were higher than in Control plants, which indicates that modified and non-modified ZnLS complexes are able to provide Zn to navy bean plants applied in nutrient solution (**Table 3**). Other authors have obtained similar results for corn, wheat and navy bean (Martín-Ortiz et al., 2009; Benedicto et al., 2011).

With respect to reactivity of modified and non-modified ZnLS complexes vs. soil components (**Figure 1**), around 70% of soluble Zn remained in solution for peat and Ca montmorillonite and 55% for illite while only 20% remained for dolomite and small amounts for lime. Modified and non-modified ZnLS complexes showed the same behavior with respect to the reactivity with different soil materials, which means this property does not depend on the wood sources or LS modifications. However, Benedicto et al. (2011) determined the stability of ZnLS complexes used in this work, as a function of pH in the presence of Ca and all the products revealed to be almost 100% soluble at pH below 7.5. This study shows the interaction with different soil materials affects the Zn solubility and it decreases as the soil pH increases. Therefore, taking into account only these results in calcareous soil conditions, where is common the zinc deficiency, the zinc LS would tend to stay retained in soil and not soluble for the plant taking up (Álvarez et al., 2001). However, the availability of this retained fraction on plant nutrition has not been yet elucidated.

The ^67^Zn tracer technique allowed monitoring Zn from the fertilizer in soil experiments and distinguishing from the native Zn contained in the soil. A combination of ^67^Zn isotope and mathematical deconvolution was an important tool to evaluate the efficacy between different treatments to correct zinc deficiency.

There were significant differences among ZnEDTA and ZnLS complexes for Zn_Fer_ and Zn_Nat_ content in navy bean leaves at 21 DAT for the first soil experiment (**Table 4**). ZnEDTA showed the highest concentration for Zn_Fer_, Zn_Nat_ and Zn_Total_. Rico et al. (1996) obtained similar results for wheat (Zn_Total_). Álvarez and Rico (2003) as well as Prasad and Sinha (1981) suggested the diffusion as the principal mechanism which contribute to plant nutrition when stable chelates as ZnEDTA are applied to calcareous soils. Singh et al. (1986) also observed the ZnEDTA efficiency for navy bean plants in calcareous soil and argued that chelating agents enhance the uptake of Zn by increasing the rate of diffusion in the immediate vicinity of plant roots.

The aim of LS chemical modification is to improve physical and chemical properties, especially zinc complexing capacity, and hence, increase LS efficiency. ZnLSEO and ZnLSEU presented the highest amounts of Zn_Total_ in leaves for the hydroponic assay (**Table 3**). We selected ZnLSEO for the first soil experiment because of its highest content in carboxylic groups would ensure higher zinc complexing capacity and thus, more efficient zinc nutrition for the plants. However, ZnLSEO didn’t show better results than non-modified ZnLS complexes (**Table 4**). Benedicto et al. (2011) concluded that a high zinc complexing capacity did not necessarily imply a high Zn supply to plants in hydroponic assays. Moreover, ZnLSEO pH was 6.8 (**Table 1**), the soil pH was 7.9 and the nutrient solution used for the irrigation had a pH between 8.0 and 8.2. Benedicto et al. (2011) observed that ZnLSEO solubility decreased almost 20% at pH 8.5 in calcareous conditions. Thus, almost 80% of ZnLSEO remained soluble in the calcareous soil and its efficiency was lower than non-modified ZnLS.

Taking into account those results, a second soil experiment was designed and different doses (1.5, 2.3, 4.6 μmol ^67^Zn per pot) of non-modified ^67^ZnLS (ZnLSE and ZnLSS) were compared respect to ^67^ZnEDTA (2.3 μmol ^67^Zn per pot).

In this last soil experiment, ZnEDTA showed, as the first experiment, the highest content of Zn_Total_ in leaves (**Figure 2**). Moreover, for soluble Zn_Fer_ and Zn_Nat_ in soil, ZnEDTA showed higher contents than ZnLS complexes (**Figure 3**) in pots with and without plants. This was an expected result for Zn_Fer_ caused by the high solubility of the chelates but not for Zn_Nat_, as it was almost three times larger than control. These results are apparently contradictory with theoretical models. According to Lindsay (1979), once ZnEDTA releases the zinc to the plant, EDTA should primarily chelate calcium and not zinc. This was also confirmed by López-Rayo et al. (2015) that obtained the lowest ZnEDTA presence in oxidizing conditions (pe + pH: 18) when its behavior was modeled using the software VMINTEQ 3.0. The pH decrease at the rhizosphere may explain this apparent contradiction. EDTA tends to complex zinc instead of calcium ought to the rhizosphere pH can differ in two units less than the bulk soil (Neumann and Römheld, 2012). Furthermore, soluble Zn_Nat_ amounts in pots with plants were four times larger than pots without plants. It is clear that roots play an essential role in the solubility of Zn in soil. Respect LS interactions with other nutrients, no differences were observed for micronutrients (Supplementary Tables S4–S8) but total phosphorous concentration decreased in leaves. This possible antagonism between phosphorous and zinc is surely explained by the precipitation of Hopeite [Zn_3_(PO_4_)_2_.4H_2_O] in the soil (Lindsay, 1979) formed by binding of the soluble zinc LS and soil phosphates in that pH condition.

**FIGURE 4:**
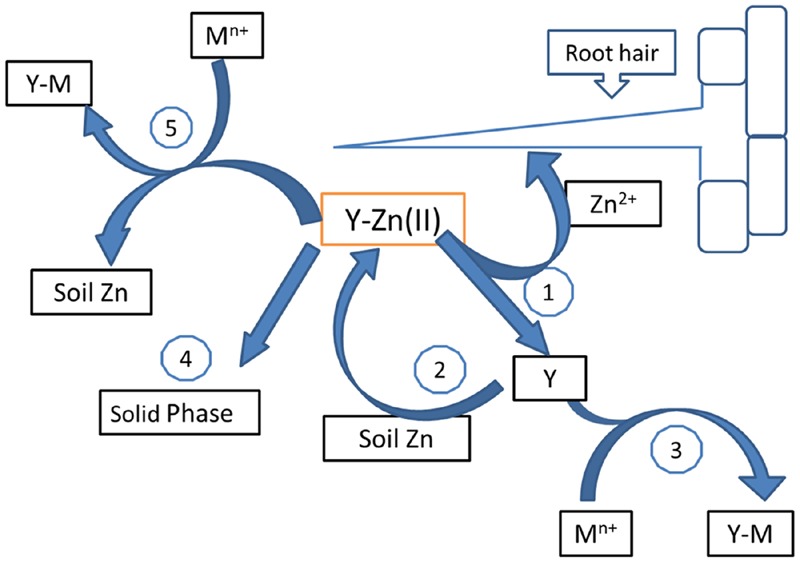
**Schematic representation of the reactions of a chelate in soils (adapted from Lucena, 2006).** (1) Zn^2+^ and Y (chelating agent) release; (2) Y binds to soluble native Zn^2+^; (3) Y complexes others soil metallic cations (M^n+^); (4) Y react with the solid phases and extract more native Zn^2+^ from the soil. (5) Isotopic exchange between Y/Zn_Fer_ and Y/Zn_Nat_.

**Figure 4** shows the proposed shuttle model for zinc chelates in calcareous soil. Zinc chelate (ZnY) applied to a calcareous soil, dissociates to attain equilibrium with Zn^2+^ and Y (1). The roots may take Zn^2+^ and Y, the chelating agent, dissolves native zinc from the soil, chelates it and carries it to the roots by diffusion (2), and the process begins again. This is the “Shuttle effect.” Also, ligand Y can chelate another cations present in soil, e.g., Ca^2+^, Fe^3+^, Fe^2+^, Cu^2+^, Mn^2+^ (3). Ligand Y can be retained by the soil surface too (4), or can react with the solid phases and extract more native zinc from the soil. Our results attempt to prove this mechanism for ZnEDTA, using ^67^Zn for this task.

Thus, this theoretical model explains ZnEDTA mechanism in the soil experiments. Steps 2 and 5 are carried out in pots experiments with plants and ZnEDTA provide Zn_Nat_ to the plants as follow:

1. Shuttle effect: Chelate releases Zn^2+^. The roots take up Zn^2+^. EDTA chelates the native Zn (step 2, **Figure 4**):

$$\begin{array}{c} {\mathrm{Zn}}_{\mathrm{Fer}}\mathrm{EDTA}⇆{\mathrm{Zn}}_{\mathrm{Fer}}+EDTA\\ \mathrm{EDTA}+{\mathrm{Zn}}_{\mathrm{Nat}}⇆{\mathrm{Zn}}_{\mathrm{Nat}}\mathrm{EDTA} \end{array}$$

Then, Zn_Nat_EDTA releases the Zn_Nat_ near the roots.
2. An isotopic exchange is also possible (step 5, **Figure 4**):

$${\mathrm{Zn}}_{\mathrm{Fer}}\mathrm{EDTA}+{\mathrm{Zn}}_{\mathrm{Nat}}-soil⇆{\mathrm{Zn}}_{\mathrm{Nat}}\mathrm{EDTA}+{\mathrm{Zn}}_{\mathrm{Fer}}-soil$$

However, the ratio between the soluble fractions (Zn_Nat_EDTA)/(Zn_Fer_EDTA) in the soil in pots with plants was 7.6:1 while in soil without plants was 4.4:1, indicating that a shuttle effect of the EDTA may occur when plants are present. In fact, root presence is the drive force in this mechanism because enables chelate agent mobility and zinc transport. In this way, ZnEDTA is more efficient than ZnLS treatments because it provides Zn_Fer_ and an extra Zn_Nat_ to the plants.

Regarding LS, although ZnLSE3 and ZnLSE2 demonstrated to be as efficient as ZnEDTA to provide Zn_Fer_ to the navy bean plants (**Figure 2**), the third dose is statistically the best to obtain the similar results to ZnEDTA fertilization (**Table 6**). Moreover, significant differences for Zn_Fer_ in leaves between treatments were observed (**Table 6**) and ZnLS from eucalyptus wood showed to be more efficient than from spruce. It is clear that Zn_Total_ contents for plants treated with ZnLS were lower than with ZnEDTA. The likely explanation is that shuttle effect and isotopic exchange are occurring to a higher extent with EDTA than with complexes. Lignosulfonate complexes are less stable and mobile than chelates in soil because of their weak bonds and high molecular weight. Therefore, ZnLS complexes do not provide extra Zn_Nat_ as synthetic chelate and the schematic representation for LS (**Figure 4**) only would show reactions 1, 3, and 4. While ZnEDTA was the most efficient treatment in providing Zn_Total_ to the navy plants, eucalyptus lignosulfonate at the maximum dose (ZnLSE3) was the most efficient treatments to supply Zn_Fer_. Further, the average molecular weight of eucalyptus lignosulfonates (LSE) is four times less (**Table 1**) than spruce lignosulfonate (LSS), hence LSE mobility is faster than LSS. Based on our results, an equal or double dose of ZnLSE applied in soil would provide similar Zn_Fer_ content in leaves to ZnEDTA.

Other authors have indicated that LS are more reactive than chelates and remain retained in soil surfaces when they were applied as a zinc source, allowing zinc to be available but non-soluble, to plant requirements (Rico et al., 1996; Álvarez et al., 2001; Álvarez and Gonzalez, 2006). In this work, we observed similar soluble Zn_Fer_ contents for ZnLS as ZnEDTA and better Zn_Fer_ availability for ZnLS than for the synthetic chelate at the highest dose, independently of the lignosulfonate source.

According to **Table 7**, navy bean fertilized with the lowest dose of ZnLSE took up similar Zn_Fer_ percentage than plants fertilized with ZnEDTA. Moreover, plants fertilized with ZnLSE took up around 4% Zn_Fer_ more than plants fertilized with ZnLSS. It is noticeable that while ZnLSE tends to improve the Zn_Fer_ distribution mainly in the plant, ZnLSS tends to remain available in soil. Chelation by low-molecular-weight organic substances is a factor that strongly affects the concentration of micronutrient cations in the soil solution and their transport to the root surfaces by mass flow and diffusion. In nutrient solution experiments, the rate of uptake of metal cations from metal–organic complexes is lower than from free cations and decreases with the size of the organic ligand (Rengel, 2012). In this case, molecular weight of spruce lignosulfonate is four times larger than eucalyptus ones, so, its mobility in the rhizosphere by diffusion may not be favored. Benedicto et al. (2011) have obtained similar results for ^67^Zn foliar applications with LS.

In relation to the results obtained in this work, ZnEDTA is the most efficient fertilizer in providing zinc to the navy bean plants (*Phaseolus vulgaris L* c. v. Negro Polo) not only Zn_Fer_ but also extra Zn_Nat_, as consequence of a shuttle effect and an isotope exchange. Furthermore, a double dose of non-modified ZnLS complex coming from eucalyptus woods, applied in calcareous soil would provide the similar Zn_Fer_ content in leaves to ZnEDTA. Moreover, ZnLSE would supply similar soluble Zn_Fer_ content in soil to ZnEDTA and higher available Zn_Fer_ than ZnEDTA, which allow the plants to take zinc along the entire growth process. Finally, ZnLSE complex is an eco-friendly and low cost option to the synthetic chelates to provide zinc to navy bean plants that grown in calcareous soil.

## Author Contributions

AB carried out the lignosulfonate characterization and the hydroponic experiments with the subsequent analysis and statistical analysis. MC carried out the soil experiments, analyzed the plant and soil material, did the mathematical deconvolution and performed the statistical study. LH-A and JL conceived the study. MC wrote the manuscript together with the revision of JL. JL designed the manuscript and supervised all the experimental work presented. All the authors read and approved the final manuscript.

## Conflict of Interest Statement

The authors declare that the research was conducted in the absence of any commercial or financial relationships that could be construed as a potential conflict of interest.

## Abbreviations

AAS: atomic absortion spectrometry
ICP-MS: inductively coupled plasma mass spectrometry
HEPES: N-(2-Hydroxyethyl) piperazine-N′- (2-ethanesulfonic acid)
ZnCC: zinc complexing capacity
ZnEDTA: zinc ethylenediaminetetraacetate
Zn_Fer_: zinc from the fertilizer
ZnLS: zinc lignosulfonate
ZnLSE: zinc lignosulfonate obtained from eucalyptus wood
ZnLSEO: zinc lignosulfonate modified by oxidation
ZnLSES: zinc lignosulfonate modified by sulfonation
ZnLSEP: zinc lignosulfonate modified by phenolation
ZnLSS: zinc lignosulfonate obtained from spruce wood
Zn_Nat_: zinc from natural sources
ZnLSEU: Zinc lignosulfonate modified by ultrafiltration
