# Supplementary material for: EDTA Shuttle Effect vs. Lignosulfonate Direct Effect Providing Zn to Navy Bean Plants (Phaseolus vulgaris L ‘Negro Polo’) in a Calcareous Soil
Source: Front Plant Sci. 2016 Nov 28;7:1767. doi: 10.3389/fpls.2016.01767 (PMC5147430; doi:10.3389/fpls.2016.01767)
Supplement: Supplementary file 1 [file Data_Sheet_1.pdf]

Supplementary Material

EDTA shuttle effect vs lignosulfonate direct effect providing zn to navy bean plants (*Phaseolus vulgaris L* C. V. Negro Polo) in a calcareous soil

María Teresa Cieschi, Ana Benedicto, Lourdes Hernández-Apaolaza and Juan J. Lucena *

Correspondence: Corresponding Author: juanjose.lucena@uam.es,

Table S1: Dry weight (g pot^{-1}) of leaves for hydroponic experiment at 21 DAT

Treatments	DW (g pot^{-1})
**Control**	2.97 ± 0.15^{ns}
**ZnLSS**	2.82 ± 0.23
**ZnLSE**	2.69 ± 0.16
**ZnLSEO**	2.86 ± 0.11
**ZnLSES**	2.61 ± 0.18
**ZnLSEP**	2.77 ± 0.11
**ZnLSEU**	2.86 ± 0.17
**ZnEDTA**	3.12 ± 0.29

ns: not significant according to Duncan´s test ($p<0.05$).

Table S2: Dry weight (g pot^{-1}) in leaves for the first soil experiment at 21 DAT

Treatments	DW (g pot^{-1})
**Control**	2.03 ± 0.11^{ns}
**ZnLSS**	2.11 ± 0.12
**ZnLSE**	2.21 ± 0.11
**ZnLSEO**	2.27 ± 0.12
**ZnEDTA**	2.17 ± 0.15

ns: not significant according to Duncan´s test ($p<0.05$).

Table S3: Dry weight (DW) of leaves in second soil experiment at 21 DAT.

Traetments	DW (g pot^{-1})
**Control**	1.37 ± 0.12[ab]
**ZnLSE1**	1.23 ± 0.09[b]
**ZnLSE2**	1.30 ± 0.06[ab]
**ZnLSE3**	1.33 ± 0.17[ab]
**ZnLSS1**	1.45 ± 0.16[a]
**ZnLSS2**	1.29 ± 0.10[ab]
**ZnLSS3**	1.37 ± 0.23[ab]
**ZnEDTA**	1.48 ± 0.17[a]

In each data range, different letters denote significant differences among the treatments according to Duncan´s test ($p<0.05$).

Table S4: Micronutrient (Cu, Mn and Fe) content (nmol pot^{-1}) in the soluble soil fraction for pots with plants

Treatments	Cu	Mn	Fe
**Control**	0.26 ± 0.03^{ab}	0.15 ± 0.04^{cd}	10.4 ± 0.46^{ab}
**ZnLSE1**	0.10 ± 0.02^{d}	0.82 ± 0.05^{b}	10.1 ± 0.60^{ab}
**ZnLSE2**	0.17 ± 0.01^{c}	0.70 ± 0.04^{b}	9.00 ± 0.46^{b}
**ZnLSE3**	0.20 ± 0.00^{bc}	0.72 ± 0.04^{ab}	9.93 ± 0.46^{ab}
**ZnLSA1**	0.25 ± 0.00^{ab}	0.88 ± 0.05^{a}	9.41 ± 0.46^{ab}
**ZnLSA2**	0.26 ± 0.04^{ab}	0.23 ± 0.04^{cd}	9.13 ± 0.46^{b}
**ZnLSA3**	0.23 ± 0.02^{bc}	0.14 ± 0.04^{cd}	8.97 ± 0.6^{b}
**ZnEDTA**	0.31 ± 0.00^{a}	0.06 ± 0.04^{d}	11.0 ± 0.52^{a}

In each data range, different letters denote significant differences among the treatments according to Duncan´s test ($p<0.05$).

Table S5: Micronutrient (Cu, Mn and Fe) content (nmol pot^{-1}) in the soluble soil fraction for pots without plants

Treatments	Cu	Mn	Fe
**Control**	0.13 ± 0.00^{abc}	0.39 ± 0.01^{ns}	2.95 ± 0.19^{ns}
**ZnLSE1**	0.10 ± 0.03^{c}	0.41 ± 0.06	4.08 ± 1.40
**ZnLSE2**	0.17 ± 0.02^{a}	0.47 ± 0.02	3.19 ± 0.58
**ZnLSE3**	0.17 ± 0.01^{a}	0.49 ± 0.06	3.34 ± 0.58
**ZnLSA1**	0.14 ± 0.00^{ab}	0.70 ± 0.02	3.27 ± 0.41
**ZnLSA2**	0.13 ± 0.00^{abc}	0.69 ± 0.00	3.01 ± 0.12
**ZnLSA3**	0.12 ± 0.01^{bc}	0.65 ± 0.01	2.98 ± 0.24
**ZnEDTA**	0.13 ± 0.00^{abc}	0.59 ± 0.25	3.41± 0.12

In each data range, different letters denote significant differences among the treatments according to Duncan´s test ($p<0.05$).ns: not significant.

Table S6: Micronutrient (Cu, Mn and Fe) content (nmol pot^{-1}) in the available soil fraction for pots with plants

Treatments	Cu	Mn	Fe
**Control**	17.0 ± 0.4^{a}	149 ± 10^{ns}	120 ± 2^{ns}
**ZnLSE1**	15.6 ± 0.2^{abc}	148 ± 2	126 ± 2
**ZnLSE2**	14.3 ± 0.6^{bc}	140 ± 6	122 ± 4
**ZnLSE3**	14.1 ± 0.5^{c}	144 ± 3	122 ± 2
**ZnLSA1**	15.2 ± 0.9^{bc}	142 ± 9	118 ± 2
**ZnLSA2**	15.1 ± 0.2^{bc}	138 ± 3	118 ± 3
**ZnLSA3**	14.1 ± 0.4^{b}	138 ± 4	120 ± 4
**ZnEDTA**	15.9 ± 0.3^{ab}	154 ± 3	126 ± 3

In each data range, different letters denote significant differences among the treatments according to Duncan´s test ($p<0.05$).ns: not significant.

Table S7: Micronutrient (Cu, Mn, and Fe) content (nmol pot^{-1}) in the available soil fraction for pots with plants.

Treatments	Cu	Mn	Fe
**Control**	19.0 ± 2.8^{ns}	151 ± 23^{ab}	137 ± 16^{ab}
**ZnLSE1**	22.9 ± 0.6	172 ± 6^{ab}	157 ± 8^{ab}
**ZnLSE2**	22.3 ± 1.7	180 ± 15^{ab}	153 ± 8^{ab}
**ZnLSE3**	22.9 ± 1.6	214 ± 17^{a}	158 ± 4^{ab}
**ZnLSA1**	19.0 ± 2.1	151 ± 17^{b}	139 ± 15^{ab}
**ZnLSA2**	23.3 ± 2.4	183 ± 20^{ab}	163 ± 19^{a}
**ZnLSA3**	17.7 ± 0.2	135 ± 1^{b}	119 ± 4^{b}
**ZnEDTA**	18.3 ± 0.7	145 ± 4^{b}	127 ± 6^{ab}

In each data range, different letters denote significant differences among the treatments according to Duncan´s test ($p<0.05$).ns: not significant.

Table S8: Phosphorous concentration (g P Kg^{-1} DW) and micronutrient (Cu, Mn, and Fe) content (µmol pot^{-1}) in leaves for the second soil experiment at 21 DAT.

**Treatments**	**P**	**Cu**	**Mn**	**Fe**
**Control**	4.7 ± 0.1^{ab}	0.16 ± 0.02^{b}	2.05 ± 0.29^{ab}	2.35 ± 0.24^{ab}
**ZnLSE1**	3.7 ± 0.3^{c}	0.17 ± 0.00^{b}	1.18 ± 0.30^{cd}	1.69 ± 0.10^{b}
**ZnLSE2**	3.5 ± 0.2^{c}	0.18 ± 0.03^{b}	0.52 ± 0.10^{d}	1.69 ± 0.30^{b}
**ZnLSE3**	3.7 ± 0.1^{c}	0.28 ± 0.02^{a}	0.58 ± 0.20^{d}	1.90 ± 0.39^{ab}
**ZnLSA1**	3.9 ± 0.3^{c}	0.29 ± 0.02^{a}	0.61 ± 0.16^{d}	2.52 ± 0.24^{a}
**ZnLSA2**	3.5 ± 0.1^{c}	0.26 ± 0.01^{a}	1.09 ± 0.15^{cd}	2.49 ± 0.06^{a}
**ZnLSA3**	4.1 ± 0.3^{bc}	0.19 ± 0.02^{b}	1.73 ± 0.22^{bc}	1.71 ± 0.25^{b}
**ZnEDTA**	5.0 ± 0.2^{a}	0.20 ± 0.01^{b}	2.55 ± 0.25^{a}	2.45 ± 0.16^{ab}

In each data range, different letters denote significant differences among the treatments according to Duncan´s test ($p<0.05$).
